# VX‐765 enhances autophagy of human umbilical cord mesenchymal stem cells against stroke‐induced apoptosis and inflammatory responses via AMPK/mTOR signaling pathway

**DOI:** 10.1111/cns.13400

**Published:** 2020-05-27

**Authors:** Zhezhe Sun, Lei Gu, Ke Wu, Kankai Wang, Junnan Ru, Su Yang, Zhenzhong Wang, Qichuan Zhuge, Lijie Huang, Shengwei Huang

**Affiliations:** ^1^ Department of Neurosurgery The First Affiliated Hospital of Wenzhou Medical University Wenzhou China; ^2^ Zhejiang Provincial Key Laboratory of Aging and Neurological Disorder Research Wenzhou Medical University Wenzhou China; ^3^ Department of Neurology The First Affiliated Hospital of Wenzhou Medical University Wenzhou China; ^4^ Department of Neurosurgery Yuyao people's Hospital Ningbo China

**Keywords:** autophagy, human umbilical mesenchymal stem cells, stroke, VX‐765

## Abstract

**Introduction:**

To investigate the protective effect of VX‐765 on human umbilical mesenchymal stem cells (HUMSCs) in stroke and its mechanism.

**Materials and methods:**

Mouse models of ischemic stroke were established using the distal middle cerebral artery occlusion (dMCAO) method. The dMCAO mice were accordingly transplanted with HUMSCs, VX‐765‐treated HUMSCs, or VX‐765 + MHY185‐treated HUMSCs. The HUMSCs were inserted with green fluorescent protein (GFP) for measurement of transplantation efficiency which was determined by immunofluorescence assay. Oxygen‐glucose deprivation (OGD) was applied to mimic ischemic environment in vitro experiments, and the HUMSCs herein were transfected with AMPK inhibitor Compound C or autophagy inhibitor 3‐MA. MTT assay was used to test the toxicity of VX‐765. TUNEL staining and ELISA were applied to measure the levels of apoptosis and inflammatory cytokines (IL‐1β, IL‐6, and IL‐10), respectively. The expressions of autophagy‐associated proteins, AMPK, and mTOR were detected by Western blotting. TTC staining was applied to reveal the infarct lesions in the brain of dMCAO mice.

**Results:**

The pro‐inflammatory cytokines, TUNEL‐positive cells, and p‐mTOR were decreased while the anti‐inflammatory cytokine, autophagy‐related proteins, and p‐AMPK were increased in HUMSCs treated with VX‐765 under OGD condition. Different expression patterns were found with the above factors after transfection of 3‐MA or Compound C. The pro‐inflammatory cytokines, TUNEL‐positive cells, and infarct sections were decreased while the anti‐inflammatory cytokine and autophagy‐related proteins were increased in dMCAO mice transplanted with VX‐765‐treated HUMSCs compared to those transplanted with HUMSCs only. The autophagy was inhibited while p‐mTOR was up‐regulated after transfection of MHY.

**Conclusion:**

VX‐765 protects HUMSCs against stroke‐induced apoptosis and inflammatory responses by activating autophagy via the AMPK/mTOR signaling pathway in vivo and in vitro.

**Abbreviations**
AMPK/mTOR: AMP‐activated protein kinase/mammalian target of rapamycin
CCA: common carotid artery
dMCAO: distal middle cerebral artery occlusion
ELISA: enzyme‐linked immunosorbent assay
GFP: green fluorescent protein
HUMSCs: human umbilical mesenchymal stem cells
IL: interleukin
NF‐κB: nuclear factor‐κB
OGD: oxygen‐glucose deprivation
TNF‐α: tumor necrosis factor‐α

## 1 INTRODUCTION

Stroke is one of the leading causes of death and disability worldwide.^1^ Stroke brings serious neural damage to brain by triggering a series of pathophysiological responses including inflammatory changes.^2^ Stem cell transplantation has emerged as a novel regenerative therapy for stroke.^3^ Among the stem cell populations, human umbilical cord blood (HUCB) cells have been reported to exert anti‐inflammatory effects and secrete growth factors to promote cell survival in treatment of neurodegenerative disorders.^4^ Mannitol is a drug which can enhance the therapeutic efficacy of HUCB by facilitating the delivery of stem cells and their byproducts into ischemic brain.^5^, ^6^ However, inflammatory responses from the circulation may also permeate the brain when mannitol disrupts the blood‐brain barrier (BBB).^7^ Additionally, the central nervous system entry of HUCB cells is not necessarily required for neuroprotection in stroke if the specific cytokines they secreted could cross the BBB.^8^ Therefore, a new drug is needed to strengthen the therapeutic outcomes of stem cell transplantation in stroke. Human umbilical mesenchymal stem cell (HUMSC) is another sort of stem cell which has already been evaluated for its therapeutic effects on ischemic stroke.^9^ However, poor survival of donor cells impedes the efficacy of this promising method.^10^ Improving stem cell survival in an ischemic environment has surfaced as the key challenge for HUMSC‐mediated therapies in patients with ischemic stroke.

VX‐765 is a newly developed, selective, small molecule caspase‐1 inhibitor that can pass the BBB and reduce inflammation in vitro and in vivo.^11^ The drug has been used clinically to treat epilepsy and can be administered orally.^12^ Recent studies have shown that VX‐765 exhibits a wide range of biological effects on central nervous system diseases such as experimental autoimmune encephalomyelitis and Alzheimer's disease.^13^, ^14^ Oxygen‐glucose deprivation (OGD) is often used to mimic ischemic environment in vitro with the ability of inducing stem cell apoptosis.^15^, ^16^ To determine the potential of VX‐765 for treatment of cerebral ischemic injury, we investigated the effect of VX‐765 in OGD‐treated HUMSCs and in a rat model of stroke.

Autophagy is a catabolic event that maintains cell homeostasis in reaction to various stimuli.^17^ This self‐catabolic process occurs when two membrane vacuoles, called autophagosomes, engulf components of cells and degrade them through lysosomal mechanisms.^18^ Autophagy is critical in metabolic hunger and adaptive survival under stress for its potential in maintaining nutrient availability and energy levels in cells.^19^, ^20^ Apoptosis and autophagy are the main factors responsible for the poor survival of transplanted stem cells. Collected evidence supported that the survival of bone marrow‐derived mesenchymal stem cells could be enhanced when cell autophagy was activated by SDF‐1/CXCR4 axis.^21^ In addition, macrophage migration inhibitory factor was found to protect cells from apoptosis by regulating autophagy through AMPK/mTOR signaling pathway.^22^ Under normal conditions, AMPK can sense changes in cellular energy and activate autophagy by reducing ATP/AMP ratio, thereby indirectly activating mTOR, one of its major downstream targets.^23^ Activation of AMPK/mTOR signaling pathway may in turn stimulate autophagy and exert anti‐apoptotic and anti‐inflammatory effects.^24^

The anti‐inflammatory and anti‐apoptosis role of VX‐765 was documented in septic mice.^25^ However, no available study has clarified either the effect of VX‐765 on survival of transplanted cells or the potential relationship between VX‐765 and autophagy. Therefore, the present article intends to elucidate the possible mechanism underlying the effect of VX‐765 on HUMSC survival in OGD‐treated cells and in rats with stroke.

## 2 MATERIALS AND METHODS

### 2.1 Distal middle cerebral artery occlusion (dMCAO) model

All procedural and ethical considerations acquired approval of the Wenzhou Medical University Experimental Animal Ethics Committee. Adult Sprague Dawley rats (250‐300 g) were purchased from the Animal Center of the Shanghai Branch of the Chinese Academy of Sciences and maintained at the Experimental Animal Center of Wenzhou Medical University. Permanent dMCAO method and ipsilateral common carotid artery (CCA) occlusion were used to establish the focal cerebral ischemic stroke models. Firstly, the rats were subjected to anesthesia using 10% chloral hydrate (3.5 mL/kg, intraperitoneal injection). A vertical skin incision was made at the midline of the neck to separate the two lateral CCAs. Then, a 2‐cm incision was made between the right eyelid and the tragus, allowing the diaphragm to be removed from the exposed skull. After that, a microdrill was used to punch a 3‐mm‐diameter hole in the skull to reveal the brain. The dura mater and arachnoid were carefully peeled off using forceps under a surgical microscope. The bilateral CCA was ligated, and then, the right MCA was occluded using electrocoagulation. After 60‐minute ligation, the two lateral CCAs were released and the incision was sutured. Sham‐operated rats underwent the same procedure without occlusion of the distal MCA. Rectal temperature was controlled at 37.0 ± 0.5°C during surgery using a temperature‐regulated heating pad.

Rats were randomized to the following groups: the sham operation group, dMCAO group (dMCAO‐only rats), dMCAO + HUMSCs group (rats received dMCAO and 60 minutes later received GFP‐HUMSCs), dMCAO + VX‐765 + HUMSCs group (rats received dMCAO and 60 minutes later received GFP‐HUMSCs which were pretreated with VX‐765 for 12 hours), and dMCAO + VX‐765 + HUMSCs+MHY185 group (treated by 5 μmol/L of MHY185 for 4 hours, MCE).

### 2.2 Cell preparation and transplantation

HUMSCs were incubated in a proprietary complete medium with 5% CO_2_ at 37°C (both the cells and the medium were purchased from Cyagen Biosciences Inc). Cells exposed to OGD alone were used as apoptosis controls by serum‐free culturing under controlled anaerobic conditions in a glove box (Plas Labs 855‐AC; Plas Labs, Inc) containing a 37°C atmosphere.

VX‐765 (10 μmol/L; catalog number: HY‐13205; MedChemExpress) was added to the medium before 12 hours of OGD exposure. The use of VX‐765 herein was based on a previous study.^26^ The cells were pre‐incubated with AMPK inhibitor, Compound C (10 mmol/L, Merck Millipore) or autophagy inhibitor, 3‐MA (5 mmol/L, Sigma) in complete medium for 90 minutes under normoxic conditions. The cells were then transfected with a lentiviral construct containing a GFP expression motif. GFP‐HUMSCs were cultured in HUMSC growth medium and frozen at −80°C after expanding to the first generation. When necessary, GFP‐HUMSCs were thawed and transferred to a tube containing growth medium, followed by centrifugation at 111 *g* for 5 minutes. After the removal of supernatant, the cells were gently dispersed in 2‐3 mL of medium. The cell suspension was transferred to a 25‐cm^2^ flask, with additional medium added to reach a total volume of 4 mL. Then, the flasks were placed in an incubator at 37°C with 5% CO_2_. GFP expression in cells was confirmed by immunofluorescent GFP antibodies (1:5000, Santa Cruz Biotechnology) and nuclei stained with 4′,6‐diamidino‐2‐phenylindole (DAPI) (1:1000, Life Technologies). In the GFP‐HUMSC transplantation treatment group, cells were trypsinized with 0.05% trypsin solution for 3 minutes at 37°C before transplantation. Briefly, 1 × 10^5^ cells in 3 μL of HUMSC complete medium were transplanted to the center of the lesion using a micro‐injection needle at a delivery rate of 1 μL/min. The total number of cells in each group was the same. Rats in the other groups were only injected with phosphate‐buffered saline (PBS).

### 2.3 Enzyme‐linked immunosorbent assay (ELISA)

Levels of interleukin (IL)‐6, IL‐10, and IL‐1β in grinded brain tissues and HUMSCs were measured using ELISA kits (Beyotime) based on the manufacturer's instructions. HUMSC culture supernatant was collected and centrifuged at 500 *g* for 5 minutes, after which the supernatant was obtained and homogenized before ELISA. The absorbance value was measured at 450 nm using a microplate reader.

### 2.4 MTT assay

The HUMSCs were incubated in a 96‐well plate with 5 × 10^3^ cells per well. After primary incubation, 10 μL of MTT labeling reagent (5 mg/mL) was added to each well for another 4 hours of incubation at 37°C. DMSO (100 μL) was added to terminate the reaction at 37C° overnight. Finally, the optical density (OD) of the samples was measured at 590 nm using a microplate reader (Sectramax 190, Molecular Devices Corp.).

### 2.5 Western blotting

Cells were lysed in lysis buffer containing protease inhibitor cocktail (Roche Applied Sciences) and Halt Phosphatase Inhibitor Cocktail (Thermo Fisher Scientific). The injured cerebral cortex was micro‐dissected from brains harvested from euthanized rats and immediately frozen in liquid nitrogen. Tissue samples were homogenized in radioimmunoprecipitation assay buffer containing a mixture of protease inhibitors. Protein samples (20 μg) were separated by sodium dodecyl sulfate‐polyacrylamide gel electrophoresis (Beyotime Institute of Biotechnology, Inc) at 1.5 mA/cm^2^ for 90 minutes before being transferred to polyvinylidene fluoride membranes (Beyotime Institute of Biotechnology, Inc). The membranes were blocked with 5% skim milk in Tris‐buffered saline containing 0.1% Tween‐20 (Beyotime Institute of Biotechnology, Inc) for 1 hour and incubated with the primary antibodies at 4°C overnight. After being washed, the proteins were incubated with a suitable secondary antibody conjugated to horseradish peroxidase for 1 hour. Color development was allowed on the membranes using a chemiluminescent substrate (Beyotime Institute of Biotechnology, Inc). Finally, the brands were photographed with a ChemiDoc XRS apparatus (Bio‐Rad Laboratories, Inc, Hercules, CA, USA) and analyzed by Quantity One software (v4; Bio‐Rad Laboratories, Inc). Antibodies used in the experiments were LC3BI/LC3BII (1:1000, Cell Signaling Technology [CST]), Beclin‐1 (1:1000, Abcam), autophagy protein 5 (Atg5; 1:1000, Abcam), P62 (1:1000, Abcam), AMPK (1:1000, Abcam), phosphorylated (p)‐AMPK (1:1000, Abcam), p‐mTOR (1:1000, CST), mTOR (1:1000, CST), cleaved caspase‐3 (1:250, Abcam), Bax (1:1000, Abcam), Bcl‐2 (1:1000, Abcam), GAPDH (1:1,000, CST), B‐actin (1:1000, Abcam), and secondary antibody (1:5000).

### 2.6 TUNEL staining

Apoptosis of HUMSCs or cerebral tissues from rats was measured by TUNEL staining using a TUNEL Apoptosis Assay kit (Beyotime Institute of Biotechnology, Inc). TUNEL stained apoptotic nuclei; DAPI and fluorescein‐dUTP stained all nuclei. The apoptotic index (AI) = the number of TUNEL‐positive cells/ the total number of cells. AI was evaluated in 15 randomly selected fields.

### 2.7 TTC staining

Infarct size was assessed using 2, 3, 5‐triphenyltetrazolium chloride (TTC) staining. After the rats were deeply anesthetized and decapitated, the brains were quickly removed and manually cut into coronal sections from the head to the forehead with a scalpel. These sections were then cultured in 2.0% (wt/vol) TTC (Sigma) at 37°C for 20 minutes. Then, the brain sections were fixed in 4% paraformaldehyde (PFA) for 30 minutes at 4°C and finally photographed with a digital camera. The infarct area in each section was evaluated using ImageJ software (National Institutes of Health, Bethesda, MD, USA).

### 2.8 Neurobehavioral testing

To assess overall neurological deficits, modified neurological severity scores (mNSS) were assessed on rats 1 and 3 days after dMCAO. The test included a task portfolio that assessed the rats' ability to move, feel, reflect, and balance. The mNSS test scores ranged from 0 (normal performance) to 18 (maximum defect), with higher scores indicating severe neurological dysfunction. Rats with abnormal preoperative scores (>0) were excluded.

### 2.9 Immunofluorescence assay (IFA)

Rats were anesthetized with a lethal dose of chloral hydrate and perfused with 100 mL of saline followed by 100 mL of 4% PFA in 0.1 mol/L PBS (pH 7.6). Tissues were fixed in 4% PFA in 0.1 mol/L PBS at 4°C overnight and cryoprotected in 30% sucrose for 36 hours. Frozen sections of 10‐μm thickness were prepared and fixed in 4% PFA for 20 minutes. After being washed for 3 × 5 minutes with PBS, these sections were then permeated with 0.3% Triton X‐100 for 15 minutes and finally subjected to washing (3 × 5 minutes). After GFP transfection, transplanted HUMSCs were directly detected at 488 nm; nuclei were counterstained with DAPI. All samples were analyzed with a fluorescence microscope (BX51, Olympus).

### 2.10 Statistical analysis

Data are presented as mean ± standard deviation (SD). All values were analyzed in Prism software (GraphPad Software Inc). Unpaired Student's *t* tests were used to compare differences between two groups. One‐way analysis of variance was performed to compare differences involving three or more groups. The Kolmogorov‐Smirnov (K‐S) method was applied to check the normality of data distribution. *P* < .05 was considered statistically significant.

## 3 RESULTS

### 3.1 VX‐765 protects HUMSCs from OGD‐induced apoptosis and inflammatory responses

**FIGURE 1:**
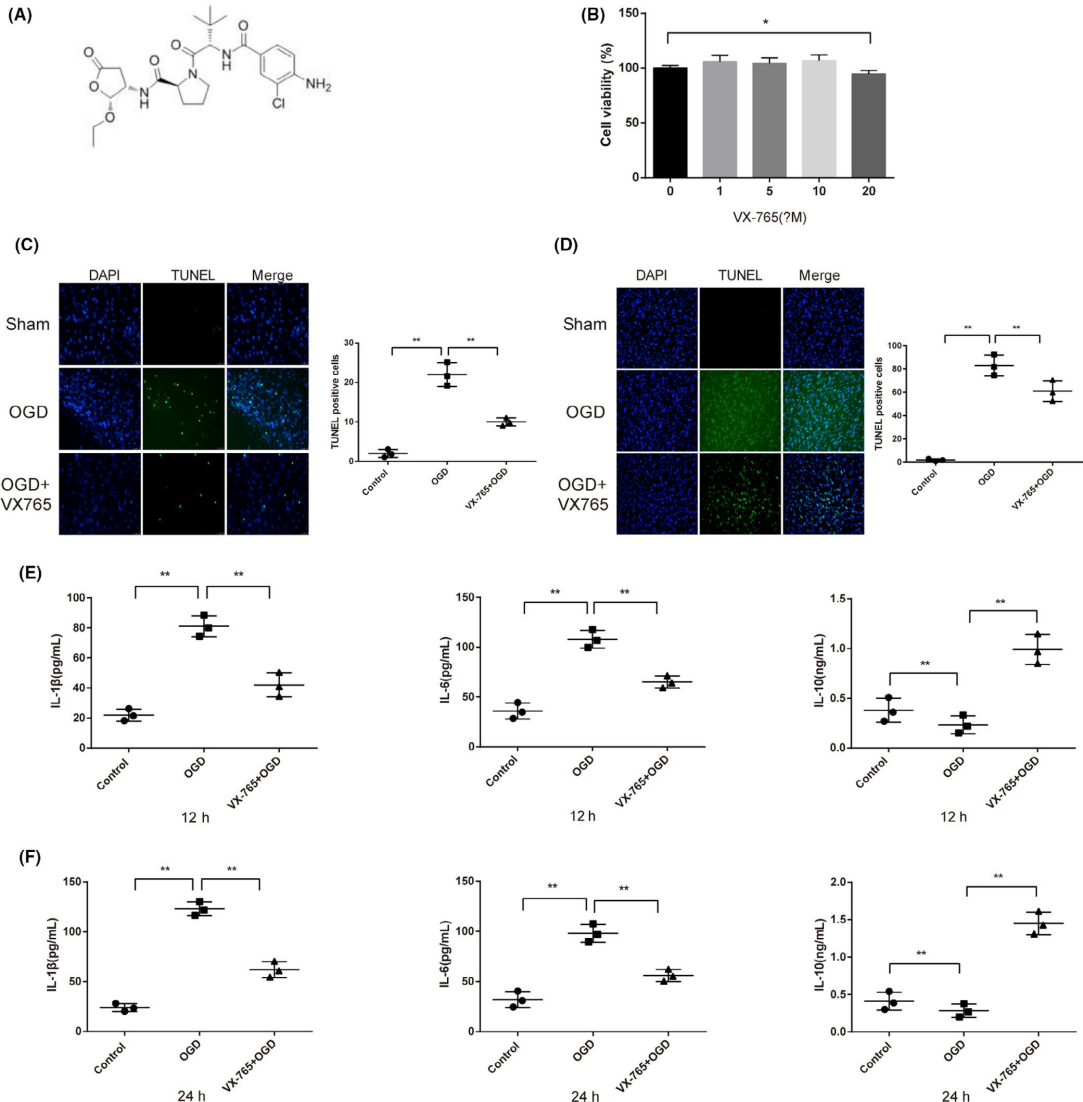
VX‐765 protects HUMSCs from OGD‐induced apoptosis and inflammatory responses. Notes: A, Molecular structure of VX‐765. B, MTT tested cell viability. C, TUNEL‐positive cells after 12 h of OGD. D, TUNEL‐positive cells after 24 h of OGD. E, ELISA measured levels of IL‐1β, IL‐6, and IL‐10 after 12 h of OGD. F, ELISA measured levels of IL‐1β, IL‐6, and IL‐10 after 24 h of OGD. (n = 4), *P < .05 **P < .01. Scale bars: 50 μm; HUMSC, human umbilical mesenchymal stem cell; OGD, oxygen‐glucose deprivation

The molecular structure of VX‐765 is shown in Figure 1A. We assessed the potential effects of different concentrations of VX‐765 on HUMSC viability. The results of MTT showed that VX‐765 ≤ 10 μmol/L did not adversely affect HUMSC viability (Figure 1B). The apoptosis of HUMSCs incubated with 10 μmol/L of VX‐765 was assessed 12 or 24 hours after OGD exposure. Compared to sham group, increases of TUNEL‐positive cells as well as pro‐inflammatory factors IL‐1β and IL‐6, and deceases of anti‐inflammatory factor IL‐10 were found in OGD groups, with the 24 hours group exceeding the 12 hours group (Figure 1C‐F). TUNEL‐positive cells, IL‐1β, and IL‐6 decreased while IL‐10 increased in VX‐765 + OGD group, compared to OGD groups (Figure 1C‐F). The above results demonstrate the anti‐apoptosis and anti‐inflammatory role of VX‐765 in HUMSCs.

### 3.2 VX‐765 promotes autophagy through AMPK/mTOR signaling in HUMSCs exposed to OGD

**FIGURE 2:**
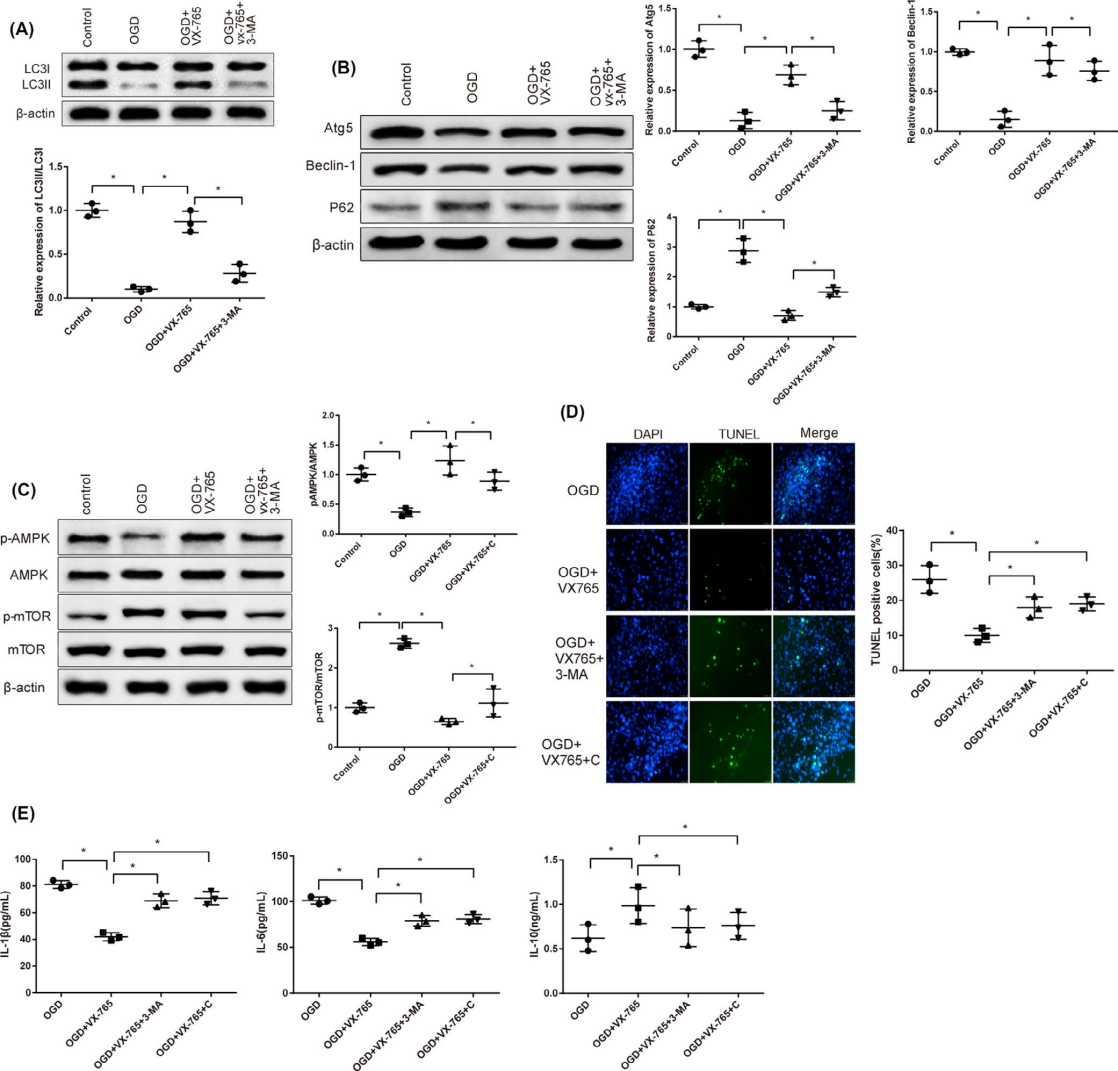
VX‐765 promotes autophagy through AMPK/mTOR signaling in HUMSCs exposed to OGD. Notes: A, B, Western blot tested the expressions of autophagy‐associated proteins. C, D, Western blot tested expressions of proteins involved in AMPK/mTOR signaling pathway. E, TUNEL staining after transfection of 3‐MA or compound C. *P < .05, **P < .01. Scale bars: 50 μm; HUMSC, human umbilical mesenchymal stem cell; AMPK/mTOR, AMP‐activated rapamycin protein kinase/mammalian target protein; OGD, oxygen‐glucose deprivation

We investigated whether 12 hours of VX‐765 treatment promoted HUMSC autophagy through activating AMPK/mTOR pathway under OGD condition and whether these phenomena could be reversed. Compared with OGD group, increased autophagy‐related proteins (Atg5 and Beclin‐1) and LC3BII/LC3BI ratio, and decreased P62 were found in VX‐765 + OGD group. Autophagy was attenuated in response to autophagy inhibitor 3‐MA (Figure 2A,B). Furthermore, the gene expressions of autophagy signaling pathway in HUMSCs were detected. Western blot analysis showed an increase of p‐AMPK and a decrease of p‐mTOR in VX‐765–treated HUMSCs. Different expression patterns were detected when cells were incubated with AMPK inhibitor Compound C (Figure 2C,D). Then, the effect of autophagy suppression on cell apoptosis and inflammation was analyzed after autophagy inhibitor 3‐MA or AMPK inhibitor Compound C was transfected into HUMSCs. TUNEL‐positive cells and expressions of pro‐inflammatory factors IL‐1β and IL‐6 increased while anti‐inflammatory factor IL‐10 decreased compared to OGD + VX‐765 group (Figure 2E). These results demonstrate that VX‐765 suppresses cell apoptosis and inflammation of HUMSCs by enhancing autophagy through AMPK/mTOR signaling pathway.

### 3.3 VX‐765 reduces apoptosis and inflammation in HUMSC‐transplanted dMCAO rats

**FIGURE 3:**
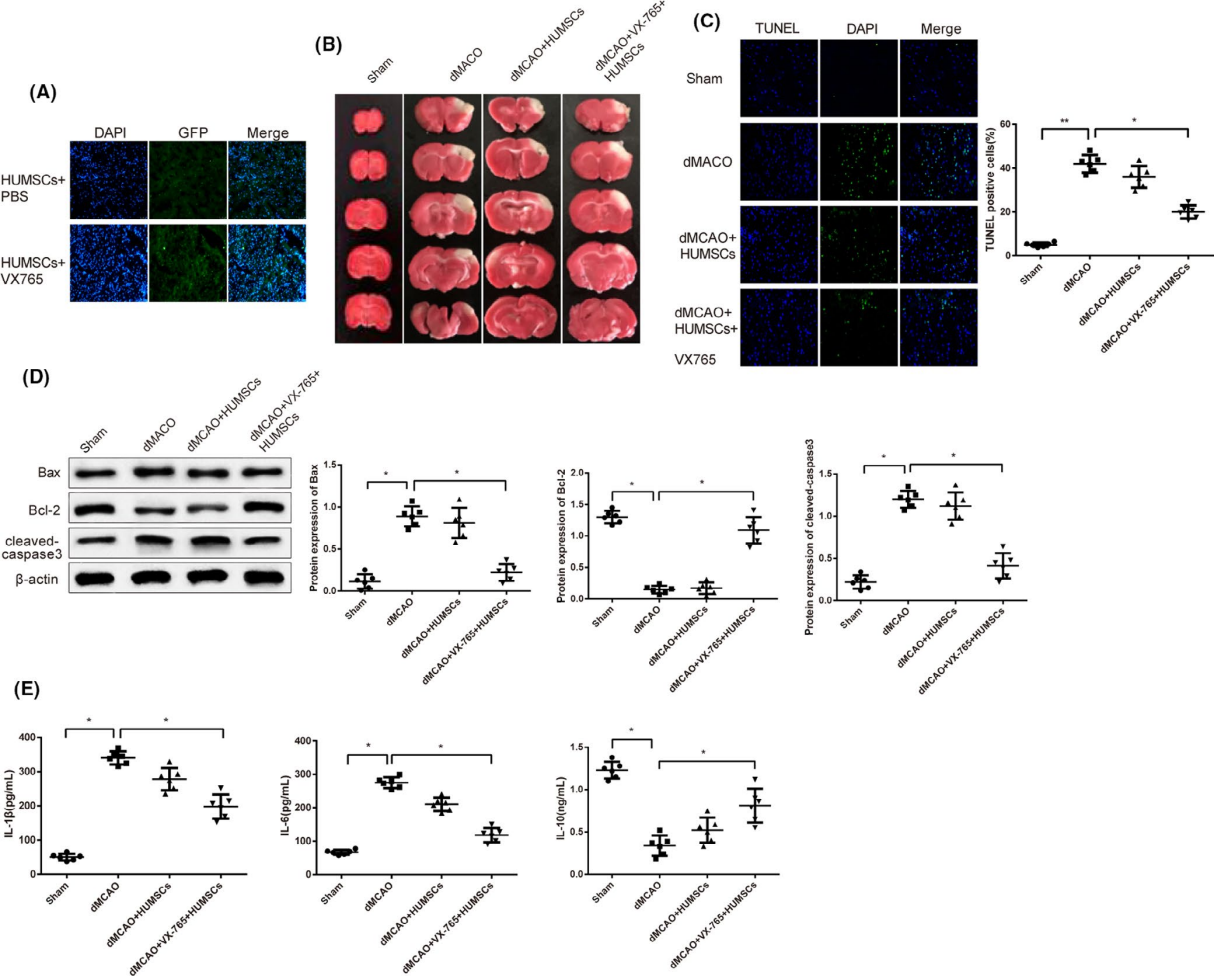
VX‐765 reduces apoptosis and inflammation in HUMSC‐transplanted dMCAO rats. Notes: A, IFA tested the intensity of GFP to verify the transplantation of HUMSCs. B, TTC measured infarcted areas of brain tissue. C, TUNEL tested apoptosis in brain tissues. D, Western blot detected apoptosis‐related proteins. E, ELISA tested the expressions of inflammatory factors. Scale bars: 50 μm; HUMSC, human umbilical mesenchymal stem cell; dMCAO, distal middle cerebral artery occlusion; IFA, Immunofluorescence assay

GFP‐stained HUMSCs were transplanted into dMCAO rats to verify the protection of VX‐765 on HUMSCs. The results of IFA demonstrated the successful transplantation of HUMSCs in dMCAO + HUMSCs group and dMCAO + VX‐765 + HUMSCs group (Figure 3A). TTC staining showed that infarcted area of brain tissue in dMCAO group was expanded compared to sham group; infracts narrowed from dMCAO group, dMCAO + HUMSCs group to dMCAO + VX‐765 + HUMSCs group (Figure 3B). Increased TUNEL‐positive cells were found in dMCAO group compared to sham group, indicating strengthened cell apoptosis; TUNEL‐positive cells decreased from dMCAO group, dMCAO + HUMSCs group to dMCAO + VX‐765 + HUMSCs group (Figure 3C). According to Western blot, the expression pattern of apoptosis‐related proteins was consistent with that of TUNEL‐positive cells (Figure 3D). ELISA demonstrated up‐regulated expressions of pro‐inflammatory factors (IL‐1β and IL‐6) and down‐regulated expression of anti‐inflammatory factor (IL‐10) in dMCAO group compared to sham group; IL‐1β and IL‐6 decreased from dMCAO group, dMCAO + HUMSCs group to dMCAO + VX‐765 + HUMSCs group while IL‐10 went the opposite (Figure 3E).

### 3.4 VX‐765 promotes autophagy of HUMSCs in dMCAO rats

**FIGURE 4:**
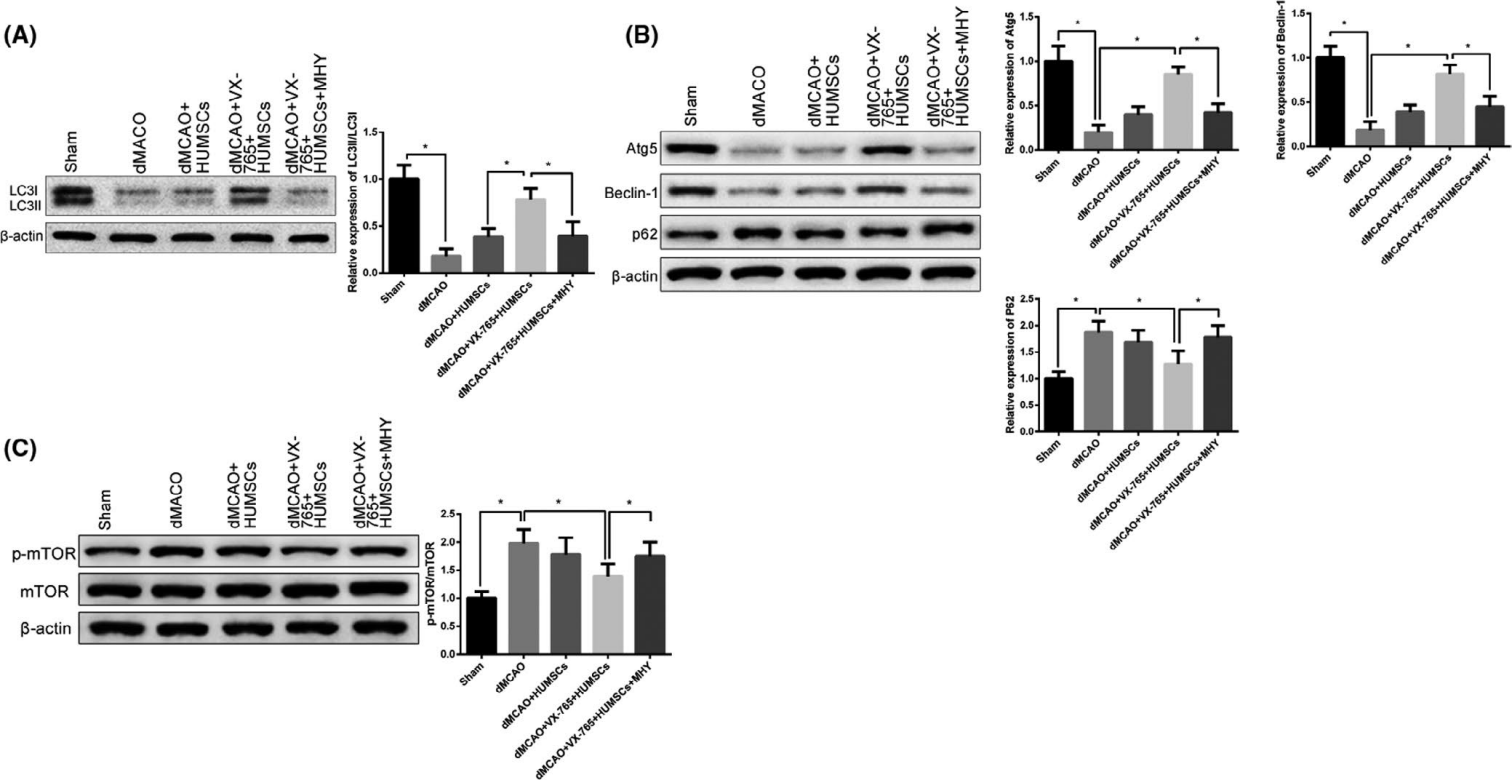
VX‐765 promotes cell autophagy in HUMSC‐transplanted dMCAO rats. Notes: A, B, Western blot tested the expressions of autophagy‐associated proteins. (n = 6). C, Western blot tested mTOR protein expression. (n = 6). *P < .05, **P < .01; HUMSC, human umbilical mesenchymal stem cell; dMCAO, distal middle cerebral artery occlusion

The expressions of autophagy‐related proteins were detected to investigate the effect of VX‐765 on HUMSC autophagy in orthotopically transplanted dMCAO rats. Compared to sham group, down‐regulation of autophagy‐related proteins (LC3II/LC3I, Atg5, Beclin‐1) and up‐regulation of P62 were found in dMCAO group; autophagy‐related proteins increased from dMCAO group, dMCAO + HUMSCs group to dMCAO + VX‐765 + HUMSCs group while decreased after transfection of mTOR activator MHY185 compared to dMCAO + VX‐765 + HUMSCs group (Figure 4A,B). Furthermore, the expression of mTOR was measured by Western blotting (Figure 4C). p‐mTOR was up‐regulated in dMCAO group compared to sham group; p‐mTOR decreased from dMCAO group, dMCAO + HUMSCs group to dMCAO + VX‐765 + HUMSCs group while increased in dMCAO + VX‐765 + HUMSCs+MHY group compared to dMCAO + VX‐765 + HUMSCs group. The above results indicate that VX‐765 regulates mTOR to enhance autophagy so as to protect HUMSCs from OGD‐induced injury.

## 4 DISCUSSION

Stroke, a highly fatal cerebrovascular disease, ranks the second leading cause of global deaths.^27^ Ischemic stroke, closely related to inflammatory responses, is the most common stroke type which results in severe neurologic disability.^28^, ^29^ Cell‐based neurovascular regeneration is a novel direction of therapies for stroke, among which autologous MSCs enjoy the most popularity.^30^ Transplantation of MSCs was reported to suppress inflammation and cell apoptosis in stroke, with the limitation of poor cell survival.^31^ To increase prospects of stem cell transplantation therapy for stroke, the present study investigated the function of VX‐765 in HUMSCs‐transplanted models in vitro and in vivo. The results demonstrate that VX‐765 reduces apoptosis and inflammatory responses by activating autophagy in HUMSCs.

First of all, we tested the expressions of inflammatory factors (IL‐1β, IL‐6, and IL‐10) and the viability of HUMSCs exposed to OGD after VX‐756 pretreatment. Increased HUMSC survival and decreased inflammatory responses were detected in VX‐765–pretreated OGD cell models. Consistent with our study, VX‐765 was found to inhibit pro‐inflammatory factors (IL‐1, IL‐18, and IL‐33) in collagen‐induced arthritis.^32^ Moreover, Do Carmo et al^33^ claimed that VX‐765 protected rat heart from ischemia‐reperfusion injury via reperfusion injury salvage kinase (RISK) pathway which involved activation of prosurvival kinases (PI3K/Akt). However, the prosurvival mechanism of VX‐765 in OGD‐treated HUMSCs was yet unknown.

In the present study, increases of autophagy‐related proteins were detected in VX‐765–pretreated HUMSCs, indicating the potential of VX‐765 for up‐regulating autophagy. Meanwhile, increased p‐AMPK and decreased p‐mTOR were detected in VX‐765–pretreated HUMSCs. Furthermore, the anti‐inflammatory and anti‐apoptosis effect of VX‐765 could be abolished by an autophagy inhibitor or AMPK inhibitor. To conclude, VX‐765 attenuates inflammation and apoptosis in HUMSCs‐transplanted OGD models by activating autophagy via AMPK/ mTOR signaling. In line with our study, with the capability of digesting pathogens and degrading damaged organelles, autophagy plays a crucial role in cell survival mechanisms under stress.^34^ A large body of evidence has shown that AMPK/mTOR participate in the progression of autophagy in numerous physiological and pathological processes. For instance, berberine prevented ox‐LDL–induced inflammation by up‐regulating autophagy through AMPK/mTOR signaling pathway.^35^ Regulation of autophagy by AMPK/mTOR pathway could also suppress endotoxemia cardiac dysfunction.^36^ AMPK and mTOR are known to function as phosphorylation regulators in post‐translational regulation of autophagy; however, mechanisms involved in autophagy regulation are found as well as in transcriptional and post‐transcriptional stages.^37^ Further research could be done to identify whether there are other autophagy regulators stimulated by VX‐765 in HUMSCs.

In in vivo experiments, VX‐765 was found to reduce infarct areas in HUMSC‐transplanted dMCAO rats. Specifically, VX‐765 alleviated apoptosis and inflammatory responses in HUMSC‐transplanted dMCAO rats by activating autophagy via regulation of mTOR. A recent study demonstrated that VX‐765 suppressed microglial pro‐inflammatory factors through inhibition of nuclear factor‐kappa B (NF‐kB) signaling thus protecting rat brain in MCAO models.^38^ Abundant research has claimed that autophagy is activated in cerebral ischemic injury.^39^ For instance, AMPK‐mediated autophagy induced by ischemic preconditioning (IPC) reduced infarct volume and cell apoptosis in pMCAO rat.^40^ Recently, many stem cells have been discovered to have therapeutic potential for ischemic stroke.^41^ MSCs are one of those promising stems cells which suppress ischemic damage by modulating inflammatory response and promoting endogenous repair in stroke.^42^ Autophagy is verified to play a pivotal role in modulating proliferation and differentiation of MSCs.^43^ The present study found that VX‐765–treated HUMSCs exerted superior protective effect on rat brain than HUMSCs alone by enhancing cell autophagy.

## 5 CONCLUSION

In summary, VX‐765 ameliorates inflammatory responses and apoptosis in HUMSCs‐transplanted models of stroke. Furthermore, we confirm that the protection of VX‐765 is achieved through up‐regulation of autophagy via AMPK/mTOR signaling pathway. Pretreatment with VX‐765 in HUMSCs facilitates the cell survival in treatment of stroke. These findings may inspire new method for improving the efficacy of cell‐based regenerative therapies for ischemic injuries.

## CONFLICT OF INTEREST

The authors have no potential conflicts of interest.
